# Analysis of gene expression changes after Latrunculin B-induced actin depolymerization

**DOI:** 10.64898/2026.10.14.759175

**Published:** 2026-10-16

**Authors:** Giorgos Kargas, Michael J. Layden, Dimitrios Vavylonis

## Abstract

Links between the cytoskeleton and gene expression are increasingly appreciated. The cytoskeleton dynamically regulates its own expression and organization through transcriptional feedback loops that sense cellular concentrations and mechanical forces. Disruption of cytoskeletal dynamics can also influence cell fate. However, the breadth, temporal dynamics, and mechanistic basis of the cytoskeleton-transcription coupling have yet to be fully resolved. To characterize this response more broadly, we re-analyzed time-resolved bulk RNA-seq data that were previously used to demonstrate that treatment with actin depolymerization drug Latrunculin B (LatB) drives differentiation of human pancreatic progenitor cells in culture. Beyond the previously identified induction of cell differentiation, we find that LatB produced extensive transcriptional changes within the actin cytoskeleton, including downregulation of actin, myosin II heavy chains, myosin I, tropomyosins, α-actinins, filamins, talin, vinculin and plastin. The changes were not restricted to the actin system, but extended to microtubule and intermediate filament systems, with noticeable changes in the expression of KIF19, MAP1A, MAP1B, Type I and II keratins and Type IV neurofilaments. More broadly, the response extended to genes involved in transcription, vesicle trafficking, metabolism, translation, ion transport, protein homeostasis, and cell signaling. The breadth of this response suggested engagement of multiple transcriptional regulatory programs. Transcription factor activity inference using decoupleR identified MRTF/SRF as the most inactivated TF network, consistent with its known role in actin homeostasis, in addition to Hippo/YAP-TEAD, MYC, stress-response and other pathways. These results provide the broader landscape of cellular response to actin depolymerization, serving as a foundation for future studies to distinguish general mechanisms of cytoskeletal homeostasis from differentiation-associated responses, and to narrow the underlying regulatory mechanisms linking cytoskeletal state to gene expression.

